# Effects of platinum/taxane based chemotherapy on acute perfusion in human pelvic tumours measured by dynamic MRI

**DOI:** 10.1038/sj.bjc.6602814

**Published:** 2005-10-18

**Authors:** K J Lankester, N J Taylor, J J Stirling, J Boxall, J A D'Arcy, M O Leach, G J S Rustin, A R Padhani

**Affiliations:** 1 Department of Medical Oncology, Mount Vernon Hospital, Rickmansworth Rd, Northwood, Middlesex HA6 2RN, UK; 2 Paul Strickland Scanner Centre, Mount Vernon Hospital, Rickmansworth Rd, Northwood, Middlesex HA6 2RN, UK; 3 Cancer Research UK Clinical MR Research Group, Institute of Cancer Research, Royal Marsden NHS Foundation Trust, Cotswold Rd, Sutton SM2 5NG, UK

**Keywords:** DCE-MRI, vascular disruptive agents, cytotoxic, *K* ^trans^, tumour

## Abstract

Dynamic contrast enhanced MRI (DCE-MRI) is being used increasingly in clinical trials to demonstrate that vascular disruptive and antiangiogenic agents target tumour microcirculation. Significant reductions in DCE-MRI kinetic parameters are seen within 4–24 and 48 h of treatment with vascular disruptive and antiangiogenic agents, respectively. It is important to know whether cytotoxic agents also cause significant acute reductions in these parameters, for reliable interpretation of results. This study investigated changes in transfer constant (*K*^trans^) and the initial area under the gadolinium curve (IAUGC) following the first dose of chemotherapy in patients with mostly gynaecological tumours. A reproducibility analysis on 20 patients (using two scans performed on consecutive days) was used to determine the significance of DCE-MRI parameter changes 24 h after chemotherapy in 18 patients. In 11 patients who received platinum alone or with a taxane, there were no significant changes in *K*^trans^ or IAUGC in either group or individual patient analyses. When the remaining seven patients (treated with a variety of agents including platinum and taxanes) were included (*n*=18), there were also no significant changes in *K*^trans^. Therefore, if combination therapy does show changes in DCE-MRI parameters then the effects can be attributed to antivascular therapy rather than chemotherapy.

Tumour vasculature is a new promising target for targeted anticancer therapies; tumour vasculature may be selectively targeted by ‘vascular disruptive’ agents, which cause rapid blood vessel shutdown (within minutes with subsequent tumour necrosis), or the development and subsequent stabilisation of tumour vasculature may be inhibited by ‘antiangiogenic agents’ (Tozer *et al*, 2002). Magnetic resonance imaging methods provide an attractive means of investigating vascular end points (and hence to evaluate the effectiveness of vascular disruptive and antiangiogenic agents) as they are widely available, noninvasive and involve no ionising radiation (Leach *et al*, 2005).

Dynamic contrast-enhanced (DCE)-MRI is the MRI method most commonly used. This involves the acquisition of a series of images over several minutes following the bolus injection of a contrast agent. Low molecular weight (<1 kDa) gadolinium chelates are usually used as contrast agents. These are small enough to diffuse out of blood vessels into tissue extravascular, extracellular spaces (EES), inducing an increase in signal intensity on T_1_-weighted MR images (Padhani and Dzik-Jurasz, 2004). The curve of signal intensity change following contrast agent injection indicates the rate of uptake of contrast agent into a tissue and its subsequent washout (Leach *et al*, 2005). Quantitative parameters relating to tissue blood flow rate, permeability surface-area product and EES volume (all of which influence the rate and magnitude of enhancement seen) are obtained from modelling of contrast agent kinetics (Tofts and Kermode, 1991; Tofts, 1997). DCE-MRI has been used to evaluate vascular disruptive and antiangiogenic agents in both xenograft studies and human trials. Significant reductions in tumour DCE-MRI kinetic parameters are seen within 4–24 h with vascular disruptive agents (Dowlati *et al*, 2002; Evelhoch *et al*, 2002; Galbraith *et al*, 2002b, 2003; Maxwell *et al*, 2002; Stevenson *et al*, 2003) and by 48 h with antiangiogenic agents (Gossmann *et al*, 2002; Jayson *et al*, 2002; Checkley *et al*, 2003a, 2003b; Morgan *et al*, 2003).

As DCE-MRI is used in early clinical trials to confirm that vascular disruptive and antiangiogenic agents target vasculature, it is important to know whether cytotoxic agents also have acute effects on vasculature. As well as aiding in the classification of agents according to their method of action, such information would also help in planning combination therapy and interpreting DCE-MRI results of such combination therapy.

The acute effects of cytotoxic agents on DCE-MRI kinetic parameters have not previously been reported, although measurements have been performed after 1–2 cycles of treatment (Barentsz *et al*, 1998; Reddick *et al*, 1999; Wolf *et al*, 2003; Ah-See *et al*, 2004). However, at these time-points objective tumour shrinkage and pathological response may also be seen, so changes in DCE-MRI parameters may be due to a reduction in blood flow secondary to tumour cell kill rather than due to direct antivascular effects. Animal studies suggest that apart from possibly the vinca alkaloids, significant acute vascular disruptive effects are unlikely (Chaplin and Hill, 2002), but longer term antiangiogenic effects of chemotherapy are recognised (Miller *et al*, 2001).

This study investigates the acute DCE-MRI effects of conventional cytotoxic agents measured 24 h after the start of the first cycle of treatment. Most patients had gynaecological cancers with pelvic or abdominal masses. All chemotherapy regimens included a taxane and/or platinum agent, as these are being used in combination therapy in current/future clinical trials. Taxanes exert their cytotoxic effects by inhibiting spindle formation but act by stabilising microtubules rather than inducing depolymerisation, so do not have acute vascular disruptive properties (Jordan *et al*, 1998; Wang *et al*, 2000). Platinums are alkylating agents that bind to DNA, inducing crosslink formation (Cavalli *et al*, 2000). Belotti *et al* (1996) found that both cisplatin and paclitaxel have antiangiogenic effects *in vitro*, but only paclitaxel had antiangiogenic effects *in vivo*.

## MATERIALS AND METHODS

Local ethics committee approval for the trial protocol and written informed consent from all participating patients was obtained. Eligibility criteria for the study were: histologically confirmed cancer at an anatomical site suitable for imaging with MRI; tumour mass ⩾3 cm in diameter; patient due to start first cycle of taxane or platinum-based chemotherapy regimen; calculated creatinine clearance >50 ml min^−1^; WHO performance status ⩽2; age ⩾18 years; no history of allergic reaction to contrast agents.

Three DCE-MRI scans were performed on consecutive days: two prechemotherapy to assess the reproducibility of the technique and one 20–24 h after the start of the first cycle of chemotherapy to assess response. Duration of taxane or platinum infusion varied from 1 h (carboplatin, docetaxel) to 6 h (cisplatin). Details of chemotherapy regimens used are given in Table 1.

The MRI studies were performed on a 1.5 T, Magnetom Symphony scanner (Siemens Medical Systems, Erlangen, Germany), using a body phased array coil. In the first scanning session, initial T_1_ and T_2_-weighted anatomical images were obtained to select four suitable contiguous slices through the centre of a tumour mass. Care was taken to place the scans in the same position on the follow-up sessions in order to obtain the same anatomical slice location. This was carried out by reference to bony landmarks, by employing the same technologist for each patient visit and by confirming acceptable anatomical relocation by the study radiologist in the quality control process prior to analysis. Proton density-weighted spoiled gradient-recalled echo (GRE) images were acquired first (echo time TE 4.7 ms, repetition time TR 350 ms, flip angle 6°, slice thickness 8 mm, four slices). Then an interleaved dynamic series of 40 T_1_-weighted GRE images were acquired (TE 4.7 ms, TR 11 ms, flip angle 35°, slice thickness 8 mm, four slices, and total imaging time 8 min 5 s) at the same slice positions. The contrast agent, gadopentetate dimeglumine (Gd-DTPA, Magnevist®, Schering Health Care Ltd, Burgess Hill, UK), was injected intravenously using a power injector (dose 0.1 mmol kg^−1^ bodyweight) at 4 ml s^−1^ during the fifth acquisition. System gain and scaling factors were maintained between acquisition of the proton density and T_1_-weighted dynamic series of images to enable the calculation of tissue contrast agent concentration (Parker *et al*, 1997).

Images were transferred to a Sun Ultra 60 workstation (Sun Microsystems, Mountain View, CA, USA) and analysed using specialist software (Magnetic Resonance Imaging Workbench (MRIW), Institute of Cancer Research, London UK) (Parker *et al*, 1998). Using information from anatomical and postcontrast T_1_ images, regions of interest (ROIs) were carefully drawn around the tumour edges for each examination by a single operator who carefully excluded areas of artefacts and blood vessels. The intra-observer variability for ROI drawing for this operator has been documented to be less than 5% (Beresford *et al*, 2005).

Quantitative analysis required conversion of the MRI signal intensities to T_1_ relaxation rates and then to Gd-DTPA concentrations following the methods described by Parker *et al* (1997). These processes are carried out in the MRIW software.

Gd-DTPA concentration at time *t*, *C*_*t*_(*t*), was calculated from the tissue *T*_1_ using the equation 
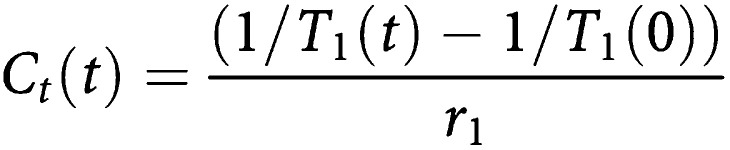
 where *T*_1_(0) is the tissue *T*_1_ without contrast and *r*_1_ is the longitudinal relaxivity of protons *in vivo* due to Gd-DTPA (taken to be 4.5 mM^−1^ s^−1^ at 1.5 T) (Donahue *et al*, 1994).

MRIW software calculates several kinetic parameters. The initial area under the Gd-DTPA concentration time curve (IAUGC – in mM s) was calculated for the first 60 s following arrival of contrast agent in the tumour. Then, the data were fitted to a standard compartmental model (Kety, 1960), to characterise the arterial influx of Gd-DTPA into the tumour EES and its venous efflux. Using this model, the time course of contrast agent concentration in tissue can be described by 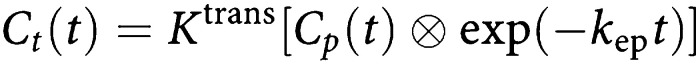 where *C*_p_(*t*) is the Gd-DTPA concentration in arterial blood plasma at time *t*; *K*^trans^ is the transfer constant for transport from plasma to the tumour EES (min^−1^), *k*_ep_ is the rate constant for transport from the EES back to plasma (min^−1^) and ⊗ denotes the convolution integral. An assumed arterial input function (AIF) was used for the modelling procedure (Weinmann *et al*, 1984; Tofts, 1997) as described previously (Galbraith *et al*, 2002a).

Values were calculated on a voxel-by-voxel basis for *K*^trans^. Voxels that did not enhance and those enhancing voxels that failed the modelling process or had values >5.0 min^−1^ were excluded from analysis. The analysis was performed on combined voxel data from all slices containing tumour, taking the median voxel value as representative of central tendency. Median rather than the mean voxel values were used as the histogram distributions of some kinetic parameters were skewed.

Data were statistically analysed using StatsDirect software (Sale, UK). The statistical analysis used to determine reproducibility has been described previously (Bland and Altman, 1996a, 1996b; Galbraith *et al*, 2002a; Padhani *et al*, 2002). The key statistical parameters are the 95% confidence intervals for change for a group of n patients and for an individual patient (the latter also known as the repeatability statistic). The 95% confidence intervals can then be used to determine whether a change in a kinetic parameter following an intervention is statistically significant or not (see below). The within-patient coefficient of variation (wCV) was also calculated. In addition, the intra-class correlation coefficient (ICC), which gives an estimate of the reliability of the measurement method, and the ratio of between-patient variance to the within-patient variance, F (obtained from a one-way analysis of variance –ANOVA), were obtained.

In summary, for each patient, the difference *d* between the two pretreatment measurements of a parameter was calculated. Data were transformed using natural logarithms if the variability of *d* was found to depend on its mean value (Bland and Altman, 1996b). The square root of the mean squared difference, dsd, (=√[(Σ*d*^2^)/*n*] where *n* is the number of patients) was then calculated. The 95% confidence interval for change for a group of *n* patients is then equal to ±(1.96 × dsd)/√*n*). For an individual patient, *n*=1 so the 95% confidence interval for change is equal to ±(1.96 × dsd), which is also known as the repeatability statistic, *r* (Bland and Altman, 1996a).

The within-patient standard deviation wSD=dsd/√2, as there are two pretreatment measurements. This is a measure of the precision of the measurement error. The difference between a patient's parameter measurement and the true value is expected to be less than 1.96 × wSD for 95% of observations. The wCV is then obtained by dividing wSD by the group mean pretreatment value for each parameter. wCV quantifies measurement error relative to the size of the (positive) kinetic parameters. If data had to be transformed, then wCV was approximated by wCV=e^wSD^−1 (Bland and Altman, 1996b).

The results of the reproducibility analysis were then used to assess whether there had been a statistically significant change in kinetic parameters due to chemotherapy, either for individual patients or the group. As there were two pretreatment measurements (days 1 and 2), the mean of the two pretreatment examinations was taken as the pretreatment value for each parameter. The null hypothesis was that there would be no difference between this combined pretreatment value and the post-treatment value, that is, there would be no changes seen at 24 h following treatment.

For individual patients, the repeatability statistic, *r*, expressed as a percentage of the group mean pretreatment value for each parameter, gives a range within which the difference between pre- and post-treatment values would be expected to lie for 95% of observations, assuming that the null hypothesis is true. If the difference falls outside this range for a particular kinetic parameter, then a significant change was deemed to have occurred. Similarly, to assess mean response in the group, the 95% confidence interval for change, expressed as a percentage of the group mean pretreatment value, gives the range required.

## RESULTS

In total, 24 female patients were imaged. The average age was 56 years old (range, 29–74 years). Data from four patients were excluded from analysis (three technical failures, one voluntary patient motion). Data from the remaining 20 patients were used for the reproducibility analysis (the two pretreatment scans). Post-treatment (day 3) data were available for 18 patients (data could not be obtained from all slices on day 3 for one patient due to internal organ motion and one patient was unable to complete the post-treatment scan, due to treatment toxicity). In total, 11 patients received a taxane or platinum agent only (numbers 1–11, Table 1). The remaining patients received a taxane and/or platinum based regimen, with the addition of other agents. The average time from the start of chemotherapy to the third scan was 21 h (range 17–23 h). Whole-group analysis regardless of the chemotherapy used was performed; those patients who received taxane or platinum chemotherapy (*n*=11) were also analysed separately for antivascular effects.

Table 1 shows patient details including diagnosis, tumour area (taken from central slice), chemotherapy regimen used and response to treatment. Most tumours had both solid and cystic components. Patient order corresponds to that given in the figures (see below). Overall response was assessed after three cycles of chemotherapy either on CT or MRI imaging or by CA125 measurements (based on RECIST or CA125 criteria) (Therasse *et al*, 2000; Rustin, 2003).

Table 2 summarises the results of the reproducibility analysis for tumours. The individual patient repeatability for tumour *K*^trans^ and IAUGC were −40.0 to +66.7%, and ±33.7%, respectively. For the whole group with day 3 data (*n*=18), the 95% confidence intervals for change (expressed as percentage of the group mean pretreatment value) were –11.9 to +13.6% and ±8.8% for tumour *K*^trans^ and IAUGC, respectively. For the subgroup receiving platinum/taxane agents only, the 95% confidence intervals for change were −15.3 to +18.1% and ±12.0% for tumour *K*^trans^ and IAUGC, respectively (NB *K*^trans^ confidence intervals are asymmetrical due to the log transformation because the difference between the two pretreatment measures was found to depend on the mean value (Bland and Altman, 1996b)).

**Figure 1:**
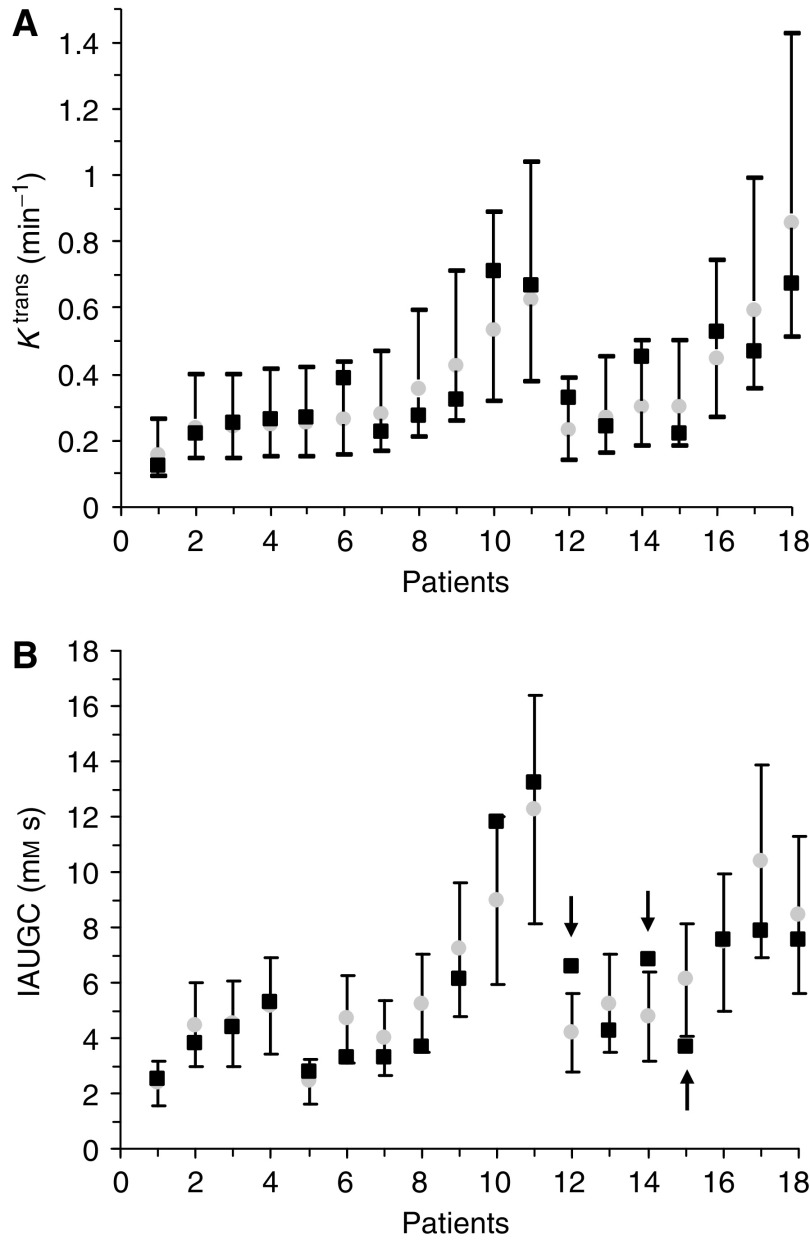
Results for individual patients. (**A** and **B**) (*K*^trans^ and IAUGC) Mean pretreatment (grey circle) and post-treatment values (black square) and the repeatability range for each parameter. Patients are ordered by mean pretreatment *K*^trans^ value, demonstrating that the repeatability range is dependent on mean pretreatment value. Patient numbered 1–11 received a platinum or taxane only. The rest received a platinum or taxane plus other chemotherapy agents (see Table 1).

Figure 1A and B show tumour pretreatment values (mean of the two pretreatment examinations), the post-treatment values and repeatability ranges for *K*^trans^ and IAUGC, respectively, for each patient (patient order corresponds to Table 1). There were no significant changes post-treatment in *K*^trans^ (Figure 1A) or in IAUGC (Figure 1B).

When data were analysed as a group (*n*=11) for the patients who received a platinum or taxane only, again there were no significant changes in *K*^trans^ or IAUGC. When all patients were included in the analysis (*n*=18), there were no significant changes in individual *K*^trans^. Two patients had significant increases (numbers 12 and 14 in Table 1: increases of 57.0 and 43.5%, respectively) in IAUGC and one patient had a significant decrease (no. 15: decrease of 39.3%). There were no significant changes in group *K*^trans^ or IAUGC.

## DISCUSSION

In this study we were very careful with patient selection to only include those who had lesions in body parts that showed little respiratory motion; this accounts for the low failure rate for our DCE-MRI examinations. This was carried out because we wanted to optimise the DCE-MRI technique and to obtain a large group of patients that received platinum and taxane-based chemotherapy. As a result, most of our patients had tumours of pelvic origin. Our patient population was therefore not typical of phase 1 clinical studies, which would ordinarily have a more heterogeneous group of patients including metastatic lung and liver lesions.

The reproducibility analysis shows that there is a wide inherent variability in individual patient repeatability for *K*^trans^ and IAUGC. This level of reproducibility has also been found in previous studies. In a phase I study of the vascular disruptive agent, Combretastatin A-4-phosphate (CA-4-P), the individual repeatability for *K*^trans^ was −44 to +79% and the 95% confidence intervals for change in 16 patients was −14 to +16%) (Galbraith *et al*, 2003). Evelhoch *et al* also performed a reproducibility analysis as part of a phase I study of ZD6126, another vascular disruptive agent. The *wCV* was ±18% for tumour IAUGC (*n*=16, scans performed 2–6 days apart, with no treatment in the intervening period) (Evelhoch *et al*, 2004). From the data given in Evelhoch's *et al* (2004) paper, the individual patient repeatability can be calculated as ±48.9% and the group 95% confidence interval for change as ±11.2% for IAUGC. However, in both these trials, significant reductions in DCE-MRI kinetic parameters were still seen. The CA-4-P study was performed at our centre and the same methods used for *T*_1_ calculation and to obtain *K*^trans^. In the CA-4-P study, there was a group mean reduction in *K*^trans^ of 29% for 16 patients 24 h following treatment with ⩾52 mg m^−2^ CA-4-P. When analysed as individuals, there were reductions in *K*^trans^ in three patients greater than the limits set by the repeatability statistic (Galbraith *et al*, 2003). In the ZD6126 study, there was a significant group mean reduction in tumour IAUGC of 38.7% (nine patients, 10 tumours, measured 6 h after administration of 56–112 mg m^−2^ ZD6126) and three of these patients had reductions in IAUGC greater than the limits set by the repeatability statistic (Evelhoch *et al*, 2004). In comparison, in our study there was no significant reduction in group *K*^trans^ or IAUGC. Given the wide within-patient variation in *K*^trans^ and IAUGC, it is not possible to state that the cytotoxic agents tested have no acute effects on tumour vascularity. However, based on the results from this study, one is unlikely to expect large antivascular effects in the acute setting due to cytotoxic agents alone. Therefore, if significant acute reductions in *K*^trans^ and IAUGC seen in combination cytotoxic and antivascular therapies, one may presume they are due to the vascular disruptive agent (or a synergistic effect with the cytotoxic agent).

Chaplin and Hill (2002) compared the acute effects of CA-4-P with a number of cytotoxic agents, including cisplatin and paclitaxel, in the murine CaNT tumour. Change in functional vascular volume (measured using Hoechst 33342) at 24 h after drug administration was used to assess vascular disruptive activity. There was a >80% reduction in functional vascular volume following treatment with CA-4-P, but no reduction following treatment with any of the cytotoxic agents (Chaplin and Hill, 2002). CA-4-P is classified as a tubulin depolymerising agent (Thorpe, 2004). The vinca alkaloids are also tubulin depolymerising agents, exerting their cytotoxic effects by the inhibition of spindle formation and subsequent mitotic arrest (Jordan *et al*, 1998). However, whereas CA-4-P induces acute blood vessel shutdown at 1/10th of the maximum tolerated dose (MTD) in an animal model (Dark *et al*, 1997), the vinca alkaloids only have sustained vascular disruptive effects at close to the MTD (Baguley *et al*, 1991; Hill *et al*, 1993, 1995; Chaplin *et al*, 1996; Sersa *et al*, 2001). Several cytotoxic agents do have a degree of antiangiogenic activity (based on *in vitro* and *in vivo* assays) (Miller *et al*, 2001) and continuous low-dose scheduling of cytotoxic agents can produce antiangiogenic effects (Browder *et al*, 2000; Colleoni *et al*, 2002; Kerbel *et al*, 2002). However, as there are no set criteria for defining antiangiogenic activity, it is difficult to establish its relative importance in comparison with an agent's cytotoxic action (Miller *et al*, 2001).

DCE-MRI, using low molecular weight contrast agents such as Gd-DTPA, is now widely available and can be incorporated into clinical trials relatively easily. *K*^trans^ has both blood flow rate and permeability components (as Gd-DTPA is not freely diffusible) and its biological meaning is dependent on the balance between capillary permeability and blood flow in the tissue of interest (Tofts, 1997; Tofts *et al*, 1999). For extra-cranial tumours, the blood flow component dominates *K*^trans^ (Tofts, 1997; Tofts *et al*, 1999) and the usage of *K*^trans^ to monitor the vascular effects of drugs is now accepted. This has been validated by Maxwell *et al* (2002), who showed that changes in *K*^trans^ (and IAUGC) in response to CA-4-P in a rat carcinosarcoma model matched changes in blood flow rate measured using uptake of ^125^I-iodoantipyrine (IAP). Moreover, the pattern and time course of changes in *K*^trans^ seen in a clinical phase I trial of CA-4-P was similar to that seen in animal models (Galbraith *et al*, 2003), indicating the appropriateness of using *K*^trans^ as a surrogate marker of vascular response to treatment. This view has been endorsed by specialist panels, meeting under the auspices of Cancer Research UK (CR-UK) (Leach *et al*, 2005) and the US National Cancer Institute (http://imaging.cancer.gov/reportsandpublications/ReportsandPresentations/MagneticResonance).

Two patients had small but significant increases in IAUGC. One (patient 14) went on to have a partial response to chemotherapy and the other (patient 12) was not evaluable for response (see Table 1). The IAUGC parameter cannot be simply related to tumour physiology, but it is a quantitative parameter that may be obtained without mathematical modelling. In our study, IAUGC correlated very strongly with *K*^trans^ (Spearman's *ρ*: 0.86, *P*<0.0001), confirming that it is an appropriate biomarker for antivascular effect. These increases do not fit into a pattern consistent with an antivascular effect (see above) and the cause for this effect is also uncertain, but could be related to increased blood flow. Griffon-Etienne *et al* (1999) reported on the use of intravital microscopy to measure red cell flux (the number of red cells passing a point per minute) and found an increase in the first few days following administration of paclitaxel or docetaxel. An increase in relative tumour blood flow rate has also been seen following cyclophosphamide and 5-fluorouracil (measured using ^86^Rubidium chloride extraction) (Braunschweiger, 1988; Li *et al*, 1991). These changes may be due to reduced blood vessel compression secondary to tumour cell kill resulting in reductions in interstitial fluid pressure (Griffon-Etienne *et al*, 1999).

There are uncertainties with regard to the reliability of kinetic parameter estimates derived from the application of tracer kinetic models to T_1_-weighted DCE-MRI data. These derive from assumptions implicit in kinetic models and those for the measurement of tissue contrast agent concentration. For example, the Tofts' model uses a standard description of the time varying blood concentration of contrast agent (the arterial input function), and assumes that the supply of contrast medium is not flow limited and that tissue blood volume contributes negligibly to signal intensity changes compared with that arising from contrast medium in the interstitial space. We have used a two-point technique (proton density and initial T_1_-weighted image) to calculate relaxation values. There are alternative schemes for calculating these values that may be more accurate. However, it should be noted that there are no head to head comparisons of such techniques. The technique that we have used has been used successfully to assess vascular response to therapy. Furthermore, international consensus meetings have recognised that this is a controversial area and have sort not to be prescriptive in this regard (Leach *et al*, 2005) and (http://imaging.cancer.gov/reportsandpublications/ReportsandPresentations/MagneticResonance). It is necessary to briefly address the issue of voxels that fail the modelling process, which occurs when the Tofts' model does not fit the Gd-DTPA concentration–time curve. This occurs principally because voxels are sited over blood vessels (100% blood volume) or are in tissues with a large blood volume. In such situations, semiquantitative parameters such as IAUGC can still be used to assess tissue enhancement and tissue response to therapy. However, as noted above, the IAUGC parameter cannot be simply related to tumour physiology; in this study, IAUGC was shown to correlated very strongly with *K*^trans^ (see above). Interested readers are invited to review the recent article by Collins and Padhani (2004), where these issues are comprehensively discussed. Despite these complexities, it is important to remind readers that quantitative kinetic parameters can provide insights into underlying tissue patho-physiological processes and it is possible to use quantitative DCE-MRI as a tool for decision making in the clinic and pharmaceutical drug development.

In summary, within 24 h after taxane/platinum-based chemotherapy regimens, no significant reductions in kinetic parameters derived from T_1_-weighted dynamic MRI are seen. However, it is not possible to state categorically that cytotoxic agents tested have no acute effects on tumour vascularity (if they are present, then they are beyond the resolution of our technique). Nevertheless, based on the results from this study, it is unlikely to expect large antivascular effects in the acute setting due to cytotoxic agents alone. Therefore, if significant acute reductions in *K*^trans^ and IAUGC seen in combination cytotoxic and antivascular therapies, one may presume they are due to the vascular disruptive agent. This is the first demonstration that cytotoxic agents differ from vascular disruptive agents in their effect on DCE-MRI parameters in humans, thus validates the use of DCE-MRI as a biomarker of targeting activity.

## Figures and Tables

**Table 1:** Patient details

**Patient number**	**Age (years)**	**Tumour type**	**Tumour size (cm^2^)** #	**Tumour site imaged**	**Chemotherapy regimen^a^**	**No. of prior chemotherapy regimens**	**Outcome**
1	55	Adeno-carcinoma, ovary	25.5	Pelvis – LR	Carboplatin AUC 6^b^	1	Stable disease^c^
2	59	Adeno-carcinoma, ovary	41.3	Pelvis – P	Carboplatin AUC 6	0	Stable diseases^c^
3	46	Adeno-carcinoma, ovary	65.4	Anterior abdominal wall – M	Carboplatin AUC 5, Paclitaxel 175 mg m^−2^	0	Stable disease^c^
4	66	Adeno-carcinoma, ovary	25.7	Para-aortic lymph nodes – M	Carboplatin AUC 5	0	Progressive disease^d^
5	73	Clear cell carcinoma, ovary	69.5	Pelvis – LR	Carboplatin AUC 6	2	Stable disease^c^
6	42	Primary peritoneal carcinoma	39	Pelvis – LR	Cisplatin 25 mg m^−2^, Docetaxel 60 mg m^−2^weekly	5	Stable disease^c^
7	62	Adeno-carcinoma, ovary	112.1	Pelvis – LR	Cisplatin 60 mg m^−2^, Docetaxel 40 mg m^−2^ weekly	1	Partial response^c^
8	45	Adeno-carcinoma, ovary	86.7	Pelvis – P	Carboplatin AUC 6	0	Stable disease^d^
9	54	Adeno-carcinoma, ovary	6.8	Pelvis – LR	Carboplatin AUC 6	2	Partial response^c^
10	46	Adeno-carcinoma, ovary	7.4	Pelvis LR	Carboplatin AUC 6	1	Stable disease^c^
11	67	Primary peritoneal carcinoma	103.4	Pelvis – LR	Carboplatin AUC 6, Paclitaxel 175 mg m^−2^	1	Partial response^d^
							
12	70	Mixed mullerian tumour/carcinosarcoma	153.6	Pelvis – P	Cisplatin 60 mg m^−2^, Doxorubicin 60 mg m^−2^	0	Not evaluable – only 1 cycle given
13	65	Mixed mullerian tumour	93.8	Pelvis – P	Cisplatin 70 mg m^−2^, Epirubicin 70 mg m^−2^	0	Partial response^d^
14	57	Adeno-carcinoma, ovary	66.2	Pelvis – LR	Docetaxel 80 mg m^−2^ day 1, Gemcitabine 1250 mg m^−2^ days 1 & 8	2	Partial response^c^
15	49	Granulosa cell tumour, ovary	45.5	Pelvis – P	Bleomycin 30 mg days 2, 8, 15, Etoposide 165 mg m^−2^days 1–3, Cisplatin 50 mg m^−2^ days 1 & 2.	0	Partial response^d^
16	59	Primitive neuro-ectodermal tumour	50.4	Pelvis – P	Cisplatin 50 mg m^−2^ days 1&2, Etoposide 150 mg m^−2^ days 1, 2, 3	0	Partial response^d^
17	66	Adenocarcinoma ovary	139.9	Pelvis – LR	Cisplatin 60 mg m^−2^ weekly, Etoposide 50 mg p.o. for every 21/28 days	4	Not evaluable – only 1 cycle given
18	52	Adenocarcinoma, endometrium	53.7	Pelvis – P	Cisplatin 60 mg m^−2^, Doxorubicin 60 mg m^−2^	0	Partial response^d^
							
19	74	Poorly differentiated carcinoma? Ovary? Primary peritoneal carcinoma	34.5	Anterior abdominal wall – M	Carboplatin AUC 5	0	Not evaluable – only 1 cycle given
20	29	Squamous cell carcinoma, cervix	4.5	Cervix – P	Cisplatin 60 mg m^−2^, Bleomycin 30 mg m^−2^, Methotrexate 300 mg m^−2^	0	Not evaluable – had surgery after 1 cycle

P.O.: orally; AUC: area under the curve; bd: twice daily.

a Chemotherapy given intravenously and repeated every 3 weeks unless otherwise stated. LR: local recurrence; P: primary; M: metastatic disease.

b Carboplatin dose calculated according to AUC (area under the plasma concentration–time curve).

c CA-125 criteria. Patients 1–11 received platinum or taxane agents only. Patients 19 and 20 had complete data sets for days 1 and 2 only.

d RECIST.

# Tumour size measured on central slice.

**Table 2:** Results of the reproducibility analysis

**Statistical variable**	***K* ^trans^**	**IAUGC**
Mean	0.39	6.36
dsd	0.26	1.09
wCV (%)	20.30%	12.10%
*r*	0.51	2.14
		
*r* (%) for individual patient	−40.0 to +66.7%	±33.7%
*r* (%) for group (*n*=11)	−15.3 to +18.1%	±12.0%
*r* (%) for group (*n*=18)	−11.9 to +13.6%	±8.8%
		
ICC	0.76	0.92
F	7.84	25.4

*K*^trans^: transfer constant; IAUGC: initial area under the gadolinium concentration time curve; mean: group mean pretreatment value; dsd: squared root of the mean squared difference; wCV: within-patient coefficient of variation; *r*: individual patient repeatability; *r (%)* individual patient: repeatability as a percentage of the mean for an individual patient; *r* (%) group: repeatability for the group of patients (*n*=11 or 18). *ICC*: interclass correlation coefficient; F: ratio of between-patient variance to within-patient variance.
